# Editorial: Epidemiology and risk factors for interstitial lung diseases

**DOI:** 10.3389/fmed.2024.1384825

**Published:** 2024-03-06

**Authors:** Niranjan Jeganathan, Tamera J. Corte, Paolo Spagnolo

**Affiliations:** ^1^Division of Pulmonary, Critical Care, Hyperbaric, and Sleep Medicine, Loma Linda University Health, Loma Linda, CA, United States; ^2^Department of Respiratory Medicine, Royal Prince Alfred Hospital, Sydney, NSW, Australia; ^3^Section of Respiratory Diseases, Department of Cardiac, Thoracic, Vascular Sciences and Public Health, University of Padova, Padua, Italy

**Keywords:** interstitial lung disease, idiopathic pulmonary fibrosis, epidemiology, prevalence, morbidity, trends, genetics, environment

In this Research Topic, we delve into a series of insightful articles that shed light on various aspects of Interstitial Lung Disease (ILD). This series of manuscripts includes a thorough examination of global ILD prevalence, morbidity, mortality, and future trends. The authors investigate the influence of genetic and various environmental factors on ILD, and examine the utilization of biomarkers, ultrasound, and radiomics for identification and outcome prediction in ILD. The scope of work further extends to the treatment landscape and biases in survival analysis. Collectively, these studies significantly contribute to enhancing our understanding of the complex spectrum of ILDs.

Prior epidemiologic studies have assessed the global burden of ILD in a limited capacity (1). In this series, Zeng and Jiang utilize the 2019 Global Burden of Disease data and demonstrate that 2.28 million men and 2.43 million women had ILD in 2019 worldwide. The age-standardized prevalence, mortality rate, and disability-adjusted life years of ILD and pulmonary sarcoidosis slightly increased from 1990 to 2019, with higher rates in men compared to women. In contrast, rates for pneumoconiosis showed a decreasing trend. Projections for global ILD trends until 2030 indicate stabilization. The study emphasizes the importance of global and country-specific initiatives to address the persistent burden of ILDs and advocates for targeted measures, particularly in regions with high prevalence and mortality rates.

The interplay between genetic predisposition and environmental exposures plays a significant role in the development and progression of ILD (2). In this series, Stanel et al. study familial pulmonary fibrosis (FPF), emphasizing its distinct characteristics, which include earlier onset, rapid progression, and limited response and/or poorer outcomes with treatment with immunosuppression. FPF is associated with heritable variants in telomere-related and surfactant-related genes, telomere shortening, and early cellular senescence. The complexity of FPF is further heightened by the interplay of genetic factors and environmental exposures, particularly air pollution. Lan et al. systematically review the impact of air pollution on ILD. Their meta-analysis demonstrates a significant association between acute exacerbation of idiopathic pulmonary fibrosis (IPF) and particulate matter (PM) 2.5, whereas associations with ozone, nitrogen dioxide, and PM10 remained uncertain. Given the limited number of available studies examining the relationship between air pollutants and ILD, there is a need for further research to comprehensively understand the intricate relationship between air pollution and ILD development and outcome. The study by Lee et al. observed a higher prevalence of firefighters in an ILD cohort compared to the general population suggesting an association between firefighting and ILD and emphasizing the need for systematic exposure assessments in patients with ILD. Shull et al. conducted a cross-analysis of telomere length and PM2.5 exposure in a cohort of patients with fibrotic ILD. Although no correlation with telomere length was found, this study offers insights for innovative methodologies in understanding the development and prognosis of pulmonary fibrosis.

Identifying high-risk individuals for ILD is crucial for early diagnosis and intervention and involves a combination of clinical assessments, biomarkers, and radiological tools. Li et al.'s cross-sectional study compared clinical features of systemic sclerosis (SSc) in a Chinese patient cohort with and without ILD, identifying significant factors associated with ILD in SSc. The study demonstrates the importance of recognizing specific clinical and laboratory markers for predicting ILD in SSc patients, providing valuable insights for early diagnosis and intervention. Wang et al. explored the role of serum biomarkers and lung ultrasound in connective tissue disease-related ILD (CTD-ILD). The researchers observed significantly elevated levels of B-cell activating factor levels and Krebs von den Lungen-6 levels in CTD patients compared to healthy controls, with even higher levels in those with fibrotic ILD. The study also revealed correlations between these biomarkers and severity of CTD-ILD assessed by lung ultrasound B-lines, and high resolution computed tomography chest. These findings highlight the potential utility of these markers in managing CTD-ILD. A proof-of-concept study by Venerito et al. hints at the transformative potential of radiomic analysis, beyond conventional clinical parameters to predict mortality in patients with rheumatoid arthritis-associated ILD. The study identified five radiomic features associated with mortality in RA-ILD patients, suggesting that radiomics may serve as a valuable digital biomarker for predicting outcomes and therapeutic response in ILD.

Over the past decade, antifibrotic drugs like pirfenidone and nintedanib have been approved to slow disease progression in fibrotic ILD (3). However, concerns about adverse events and skepticism about the efficacy of antifibrotics still exist in the medical community (4). The research study by Tomasetti et al. aims to provide insights into the real-world clinical experience of IPF by reporting data from a 15-year period. Analyzing a cohort of 634 IPF patients diagnosed between 2002 and 2016, the study found an overall median survival of 4.7 years, with a decline in mortality observed after 2012. The findings suggested that the year 2012 marked a turning point, coinciding with the introduction of antifibrotic treatment, the discontinuation of immunosuppressive drugs and advanced diagnostic techniques like transbronchial lung cryobiopsy associated with improved survival. The study also highlighted the positive impact of antifibrotic treatment on reducing the risk of acute exacerbations and hospitalizations. However, retrospective studies such as this are not without bias. Zheng et al. describe the importance of recognizing the impact of immortal time bias in observational studies examining associations between antifibrotic therapy and survival in patients with IPF. The results indicate that using time-fixed and exclusion methods tends to overestimate the effectiveness of antifibrotic therapy in reducing the risk of all-cause mortality, with the time-dependent method identified as the most optimal approach for minimizing this bias. The study emphasizes the importance of appropriate statistical methods in future IPF research to ensure accurate estimations of treatment effects and improve clinical decision-making.

ILDs lead to various symptoms such as shortness of breath, cough and fatigue, which significantly impact the quality of life of affected individuals (5). However, previous studies have not explored the impact of thoracic pain in ILD. The study by Scherer et al., noted that thoracic pain is a prevalent symptom in chronic ILD, especially in pulmonary sarcoidosis, and is associated with further respiratory limitations and worsened hypoxemia in advanced disease stages. The study also discusses the impact of thoracic pain on mental wellbeing, and the potential for early intervention to enhance patients' quality of life.

Many patients turn to internet resources for health information, particularly for rare diseases. Previous studies with IPF found that internet information on IPF is often incomplete and inaccurate (6). Buschulte et al. analyzed the reliability and content of information on sarcoidosis available on the internet, highlighting the need for comprehensive and accurate resources. The study stresses the importance of collaboration between healthcare professionals and patients to enhance the comprehensibility and reliability of information available to individuals' seeking resources on ILD.

As we navigate this multifaceted landscape of ILDs, the studies published in this research collection collectively contribute to a more comprehensive understanding, paving the way for improved diagnosis, management, and, ultimately, better outcomes for patients grappling with this challenging pulmonary condition.

## Author contributions

NJ: Writing – original draft, Writing – review & editing. TC: Writing – review & editing. PS: Writing – review & editing.
